# Exploration of scalp surface lipids reveals squalene peroxide as a potential actor in dandruff condition

**DOI:** 10.1007/s00403-016-1623-1

**Published:** 2016-02-03

**Authors:** Roland Jourdain, Alain Moga, Philippe Vingler, Charles el Rawadi, Florence Pouradier, Luc Souverain, Philippe Bastien, Nicolas Amalric, Lionel Breton

**Affiliations:** L’OREAL Research and Innovation, Aulnay-sous-Bois, France; Synelvia SAS, Labège, France; L’OREAL Research and Innovation, Saint-Ouen, France

**Keywords:** Dandruff scalp, Sebum, Lipids, Oxidative stress, Squalene peroxide, *Malassezia*

## Abstract

Dandruff is a common but complex disorder with three major contributing factors: (1) individual predisposition, (2) scalp sebum and (3) *Malassezia* yeast colonization. To obtain further insights into the role of sebum in dandruff biogenesis, we analyzed scalp lipid species in a cohort of ten dandruff-free (control) and ten dandruff-afflicted volunteers by gas chromatography coupled to mass spectrometry. Lipid peroxidation levels and biochemical markers of oxidative stress were also assessed. Squalene, a major sebum component, was significantly more peroxidized in dandruff-affected scalps, resulting in significantly higher ratios of squalene monohydroperoxide (SQOOH)/squalene. This was observed when comparing dandruff-affected zones of dandruff subjects to both their non-affected zones and control subjects. In addition, other biomarkers such as malondialdehyde indicated that oxidative stress levels were raised on dandruff scalps. Surprisingly, differences regarding either free or bound fatty acids were fairly rare and minor. Certain novel findings, especially squalene peroxidation levels, were then confirmed in a validation cohort of 24 dandruff-affected subjects, by comparing dandruff-affected and non-dandruff zones from the same individuals. As SQOOH can induce both keratinocyte inflammatory responses and hyperproliferation in vitro, we hypothesized that increased SQOOH could be considered as a new etiological dandruff factor via its ability to impair scalp barrier function. Our results also indicated that *Malassezia* could be a major source of squalene peroxidation on the scalp.

**Electronic supplementary material:**

The online version of this article (doi:10.1007/s00403-016-1623-1) contains supplementary material, which is available to authorized users.

## Introduction

Dandruff and scalp seborrheic dermatitis (SD) are common scalp flaking disorders affecting millions of adults worldwide [1, 14, 43], as evidenced by the 17 % of French adults who report excessive scalp flaking [23]. On the scalp, SD is characterized by large, yellow, greasy scales covering erythematous scalp areas with varying degrees of pruritus, and can also affect between 1 and 3 % of immune-competent adults [7, 17, 32, 39]. Dandruff is a less severe condition characterized by diffuse or localized patches of fine, loosely adherent white or gray flakes (corneocyte aggregates), on hair-bearing scalp areas [14]. It is now commonly accepted that both dandruff and scalp SD belong to a continuum of scalp afflictions [37]. Since the identification of a bottle-shaped fungus by Rivolta, *Malassezia* and Sabouraud in the late nineteenth century [38], etiological advances have led to the identification of three major factors in the appearance of dandruff: individual predisposition, sebaceous gland secretions and *Malassezia* scalp colonization [39].

Even less explored than the role of *Malassezia*, and especially, of *M. restricta* in dandruff etiology [6, 42], the importance of sebum is fairly evident. Firstly, dandruff is often associated with the onset of puberty and a concomitant increase in scalp sebaceous gland activity [37]. Secondly, as all *Malassezia* species except *M. pachydermatis* require exogenous lipids as nutrients, they are frequently associated with sebum-rich cutaneous areas, such as the scalp [16]. For *M. globosa*, this lipid dependence is due to its genomic deficiency in fatty acid synthase balanced by an elevated numbers of lipases [7, 44]. Thirdly, topical application of 7.5 % oleic acid, a sebum component, induces dandruff-like symptoms on the seemingly normal scalps of anti-dandruff-treated dandruff subjects. Such an effect is not seen in non-dandruff subjects [11]. The fact that certain individuals develop dandruff, whereas others do not, even in the face of identical triggers (*Malassezia* presence, sebum), is an enduring conundrum. The most common hypothesis is an underlying deficiency in scalp barrier function in those individuals pre-disposed to dandruff [11], identified by abnormal stratum corneum ultrastructure, even in the absence of flaking [43].

Initially, irritating unsaturated free fatty acids, known as byproducts of *Malassezia* scalp lipid metabolism were thought to cause dandruff [11, 37]. Subsequent investigations revealed that neither saturated nor unsaturated free fatty acids perturbed keratinocyte function [18]. However, some sebaceous free fatty acids such as lauric and sapienic acids have been described as “antimicrobials”, and could thus modify the scalp microbiome [12]. Otherwise, by measuring lipoperoxide generation, particularly squalene peroxide, *Pityrosporum ovale*, a representative of the *Malassezia* genus, was shown to have an oxidative effect on a culture medium supplemented with sebaceous lipids in vitro [9]. In spite of this wealth of data demonstrating sebum's role in dandruff etiology, the molecular mechanistic links remain to be elucidated [18, 39], especially with respect to the role played by sebaceous secretions, *Malassezia*-induced metabolites and the possible involvement of oxidative stress.

Therefore, we aimed to perform a large biochemical exploration of selected lipids and oxidative stress markers on normal and dandruff-afflicted scalps, in order to further investigate the biogenesis of dandruff. Specifically, lipid species belonging to the neutral scalp surface fraction, representing major enzymatic fatty acid products of synthesis pathways involving elongases (C12 to C22) and desaturases (C16 to C22), along with Δ9- and Δ6-desaturases, anteiso- and iso-branched fatty acids, the latter specific to human keratinocytes and sebocytes, were characterized [26]. In addition, we examined squalene, cholesterol, reserve fatty acids, derived from esterified forms (mono-di, triacylglycerols, wax and cholesterol esters), as well as the extent of lipid peroxidation, i.e., squalene peroxide (SQOOH) and malondialdehyde (MDA) levels, and the antioxidant defense system (vitamin E, catalase activity). During our investigation, another group demonstrated increased superoxide dismutase and catalase activities, and increased MDA levels in samples from patients with scalp SD [30], further validating our investigative objective.

In order to overcome the barriers of current scalp lipid sampling methods, we developed a reproducible, rapid and robust next-generation non-invasive sampling procedure. Arising from this new technique, specific, sensitive and replicable analytical methods were established to investigate relevant biomarkers. Using these new techniques on two different subject cohorts, we demonstrated that the most striking difference between dandruff-affected and non-affected scalp zones was the peroxidation of squalene, a major lipid component of scalp sebum.

## Materials and methods

### Subject recruitment

For this study, two independent cohorts were assessed. The first consisted of ten dandruff and ten non-dandruff (ND) healthy Caucasian volunteers (21–46 years old, median age of 40; one female) recruited in Paris, France. Subject characteristics are described in Table 1. The 10 dandruff subjects had adherent dandruff scores of ≥2.5 (scale from 0 to 5), and total (adherent + non-adherent) dandruff scores of ≥4.5 (scale from 0 to 10), based on a modified Van Abbe’s scale [41]. Selection criteria are detailed in Online Resource 1. All subjects used a prescribed neutral shampoo twice weekly for 2–3 weeks, the most recent shampoo 3 days prior to sampling. Dandruff subjects had to halt any anti-dandruff treatment at least 2 weeks before receiving the neutral shampoo. Consequently, they remained untreated for 4 weeks before the assessment visit. For the ten dandruff subjects, two dandruff zones (DZ) with ≥3.0 adherent dandruff scores were sampled for skin lipids or other scalp biomarkers, as subsequently described. For these subjects, two non-dandruff zones (NDZ) with scores ≤1.0 were similarly sampled while at a third zone, sebum casual level was measured using a Sebumeter®. Similar measurements were performed for the ten ND subjects.

**Table 1:** Characteristics of dandruff and control subjects from the exploratory cohort

	Dandruff subjects	Control subjects
*N*	10	10
Age (median and interquartile range)	40.0 (36.0–44.0)	40.0 (36.5–43.8)
Sex—female	1/10	0/10
Ethnicity	Caucasian	Caucasian
Total dandruff score (median and interquartile range)	5.50 (5.19–5.75)	0.13 (0.00–0.25)

Total (adherent + non-adherent) dandruff scores after 2–3 weeks of using neutral shampoo are reported on a scale of 0–10

The second study cohort was recruited in Lyon, France. This second cohort consisted of 24 dandruff-affected individuals (13 men and 11 women), with a median age of 43 ± 9 years, all presenting with a median dandruff severity score of 6.75 ± 1.4. For each volunteer, both non-dandruff (NDZ) and dandruff zones (DZ) were assessed. The same inclusion/exclusion criteria as for the first cohort were applied. For both cohorts, study investigators in charge of biological measurements were blinded as to basic cohort information (sex, age, etc.) and dandruff status. Both studies were conducted in compliance with the World Medical Association’s Helsinki Declaration, local regulations, and internal procedures based on ICH guidelines for good clinical practice (see Online Resource 1 for more details).

### Sample collection

**Fig. 1:**
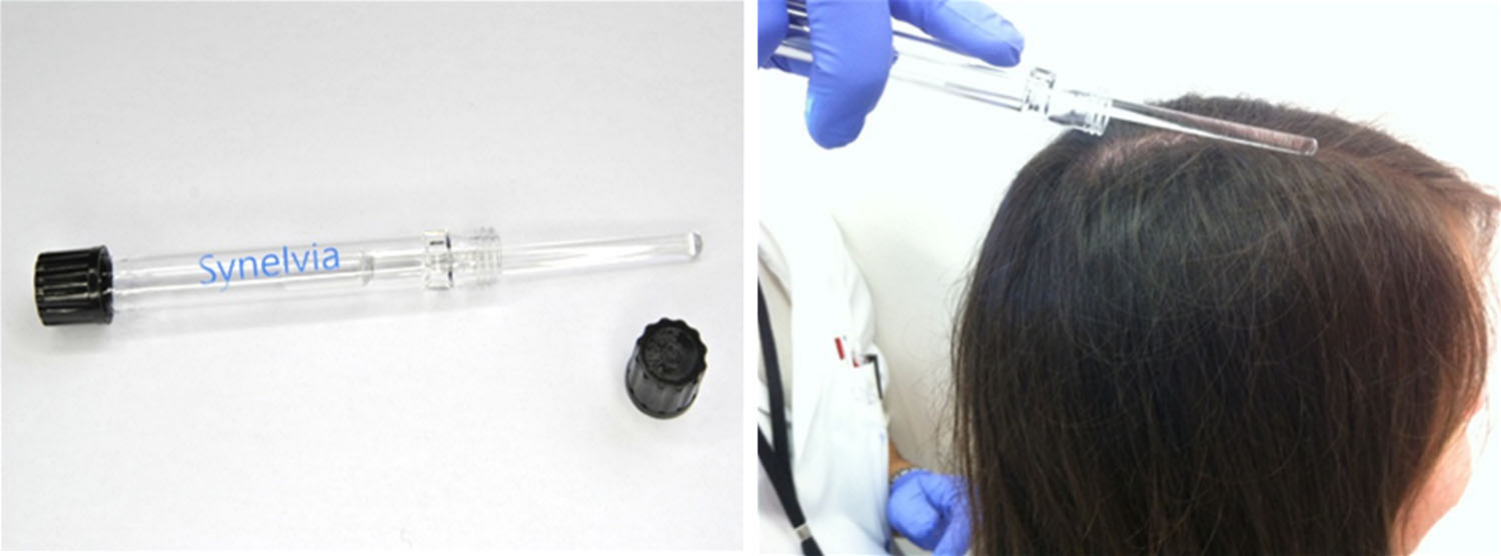
Sebum kit developed for cutaneous surface lipids sampling and its method of utilization on the scalp

A novel silica-based “sebum kit” (Fig. 1) was specifically developed for this study, based on the use of an inert sterile glass rod, specifically treated to collect lipids (Synelvia SAS proprietary method). Hair around the designated scalp sample area was cleanly parted, the rod rubbed on the sample area for 45 s, then suspended within a non-contact inert glass receptacle. Samples were stored at 4 °C until all samples were collected, then frozen at −20 °C until analysis. This sampling technique facilitates analysis of scalp surface lipids (free fatty acids, squalene, glycerides, wax and cholesterol esters, and ceramides) and lipid oxidative stress pathway markers such as SQOOH. Advantages of this non-invasive technique include ease of use, no scalp shaving, absence of solvents which can often impair analysis, and greater surface collection area resulting in larger sample volumes. In-house testing showed an absence of phthalate interference when compared to common plastic strip or tape scalp sampling methods (data not shown) [4]. The second sampling technique utilized a sterile cotton swab (ref. MW943, MWE Medical Wire) for the study of MDA, catalase, vitamin E and total proteins. After hair parting, swabs were rolled onto a 3 cm^2^ scalp area for 45 s, and then swab heads cut from the handle were placed in a stabilizing storage solution comprising a mixture of antioxidant/chelating agents (vitamin C, EDTA; Synelvia SAS proprietary method). Samples were stored at −20 °C until analysis.

### Biochemical exploration

Sebum homogenate samples were prepared in an identical manner, with subsequent specific analyses as described below. The surface scalp components covering the glass rods were then collected by vortexing the glass rods at 250*g* (VXR Basic Vibrax®, IKA®) for 1 h at ambient temperature in a water/chloroform/methanol solution (0.3/2/1), then transferred to glass tubes for subsequent analysis. This liquid/liquid extraction was performed to isolate lipids from all other markers, as cell debris can be present. Extracted samples were dried under nitrogen at 30 °C and then resuspended in a 5 ml mixture of chloroform and methanol (2/1). Total lipid extract weight was determined using a Sartorius Cubis, MSE 3.6P microbalance. For the sterile cotton swab samples, aqueous preservation solutions containing the swab heads were centrifuged twice at 20,000*g* for 10 min to collect the maximum volume of sample. Unless otherwise specified, samples were standardized and results normalized to either total lipid extract weight or total protein determined by the µBCA method (Bio Basic Inc., Amherst, NY, USA).

Neutral lipids such as the free fatty acids, squalene, cholesterol, and bound forms such as waxes, cholesterol esters and glycerides, as well as MDA, were analyzed by gas chromatography coupled to mass spectrometry [4, 15, 22] as detailed in Online Resource 1. SQOOH and vitamin E were, respectively, analyzed by either liquid chromatography coupled to mass spectrometry [13, 24] or fluorimetry [31, 36] (Online Resource 1 for details). Catalase activity was determined using the Catalase Fluorometric Detection kit (Enzo Life Sciences, Farmingdale, NY, USA).

### Statistical analysis

For both cohorts, biochemical measurements were described per zone using the median and the interquartile range (Q1, Q3). For the first cohort, biochemical measurements of ND volunteers were compared with those from both dandruff zone (DZ) and non-dandruff zone (NDZ) of dandruff-affected volunteers using the exact Wilcoxon two-sample test. For both cohorts, paired NDZ and DZ from dandruff-affected volunteers were compared using the Wilcoxon sign rank test. The two-sided significance threshold was set at 5 %. Statistical analyses were performed using SAS software 9.3 (SAS Institute Inc., Cary, NC, USA).

## Results

### Characterization of the exploratory cohort

We performed a large biochemical characterization of the dandruff scalp surface by examining a cohort of 10 non-dandruff and 10 dandruff-afflicted volunteers. Three days following the last shampoo, a scalp zone on non-dandruff ND subjects and two different zones on dandruff-afflicted individuals were assessed. The two zones on dandruff subjects (DZ and NDZ) also enabled intra-individual paired analysis. Global dandruff scores (both flaking and adherent dandruff scores) were much higher in the dandruff group, with a median dandruff score of 5.5 (5.19–5.75) compared to 0.1 (0.00–0.25) in non-dandruff individuals (Table 1). Sampling measurements and zone assessments are summarized in Table 2. Interestingly, sebum levels were found to be similar in NDZ from dandruff-afflicted subjects and in ND subjects, with measurements of 141.5 (85.0–166.0) and 124.5 (89.0–164.0) µg/cm^2^, respectively, *p* = 0.8. These results suggest that sebum levels are not a distinguishing feature of dandruff. Furthermore, total amounts of lipids (approximately 4 mg/sample) or proteins harvested were similar across the three sampled zones.

**Table 2:** Sampling data from dandruff-affected and dandruff-free control individuals of exploratory cohort

Scalp zones	Sebumetry		Lipids		Oxidative stress markers	
	Adherent dandruff scores	Values (µg/cm^2^)	Adherent dandruff scores	Weight (µg)	Adherent dandruff scores	Total proteins (mg/ml)
ND	0 (0.0–0.0)	141.5 (85.0–166.0)	0 (0.0–0.0)	4007 (3066–4309)	0 (0.0–0.0)	0.51 (0.49–0.56)
NDZ	0.5 (0.0–0.5)	124.5 (89.0–164.0)	0.5 (0.5–1.0)	4254 (3427–4675)	0.5 (0.5–0.5)	0.52 (0.49–0.66)
DZ	NA	NA	3.5 (3.0–4.0)	3954 (2343–5272)	3.5 (3.0–4.0)	0.55 (0.48–0.61)
Difference ND/NDZ	***p*** **<** **0.01**	*p* = 0.82	***p*** **<** **0.01**	*p* = 0.65	***p*** **<** **0.01**	*p* = 0.45
Difference ND/DZ	NA	NA	***p*** **<** **0.01**	*p* = 0.76	***p*** **<** **0.01**	*p* = 0.34
Difference DZ/NDZ	NA	NA	***p*** **<** **0.01**	*p* = 0.49	***p*** **<** **0.01**	*p* = 0.85

Values indicated are medians (interquartile range) and *p* values, which are only exact for comparisons between groups. Adherent dandruff scores are reported on a scale of 0–5. Sebumetry was only performed at non-dandruff-afflicted areas across the whole cohort. Significant *p* values are bolded

*ND* non-dandruff sampling area on an individual without dandruff, *NDZ* and *DZ* non-dandruff zone and dandruff zone, respectively, from an individual afflicted with dandruff, *NA* not applicable

### Biochemical exploration between dandruff and non-dandruff subjects

The quantified compounds are listed in Online Resource 2 (Table 1). As sample analysis was carried out under identical standardized conditions, the quantitative sebum composition of each volunteer was analyzed by examining neutral lipid levels via an analytical data processing lipidomics approach, where each variable was considered as independent.

**Fig. 2:**
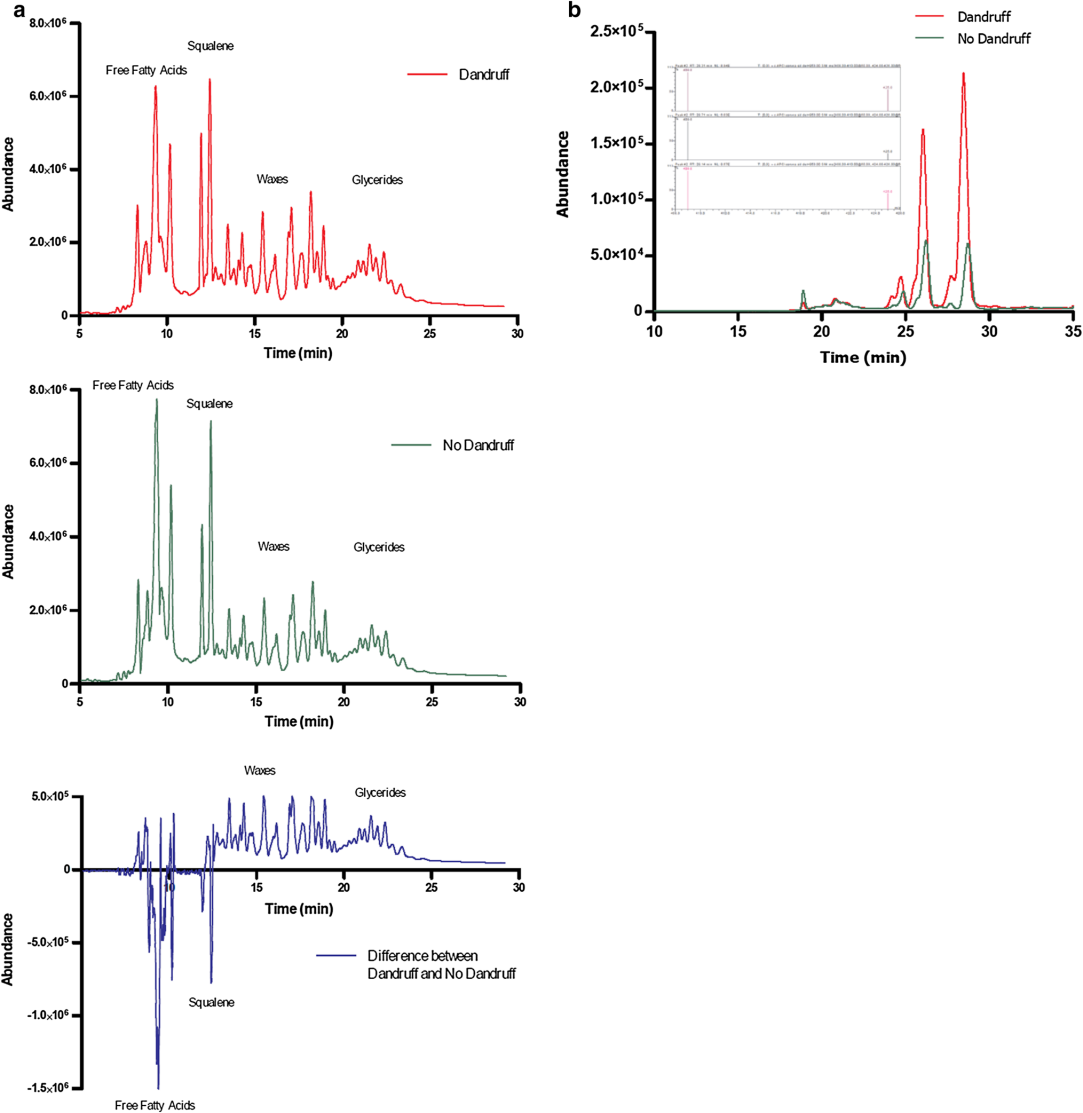
**a** Representative neutral lipid chromatographic profile, and the differential profile, of both dandruff and non-dandruff zones from a given dandruff subject. These results show reduced levels of free fatty acids and squalene with an associated increase in bound fatty acids (waxes and glycerides) at the dandruff zone. **b** Representative chromatographic analysis of SQOOH profiles at higher resolution demonstrating greater SQOOH levels at the dandruff zone compared to the non-dandruff zone from a given dandruff subject. The chromatographic profile highlights several isomeric peaks of which there are two major peaks. The mass spectra obtained for each peak is similar, confirming that these are three monohydroperoxide positional isomers

**Fig. 3:**
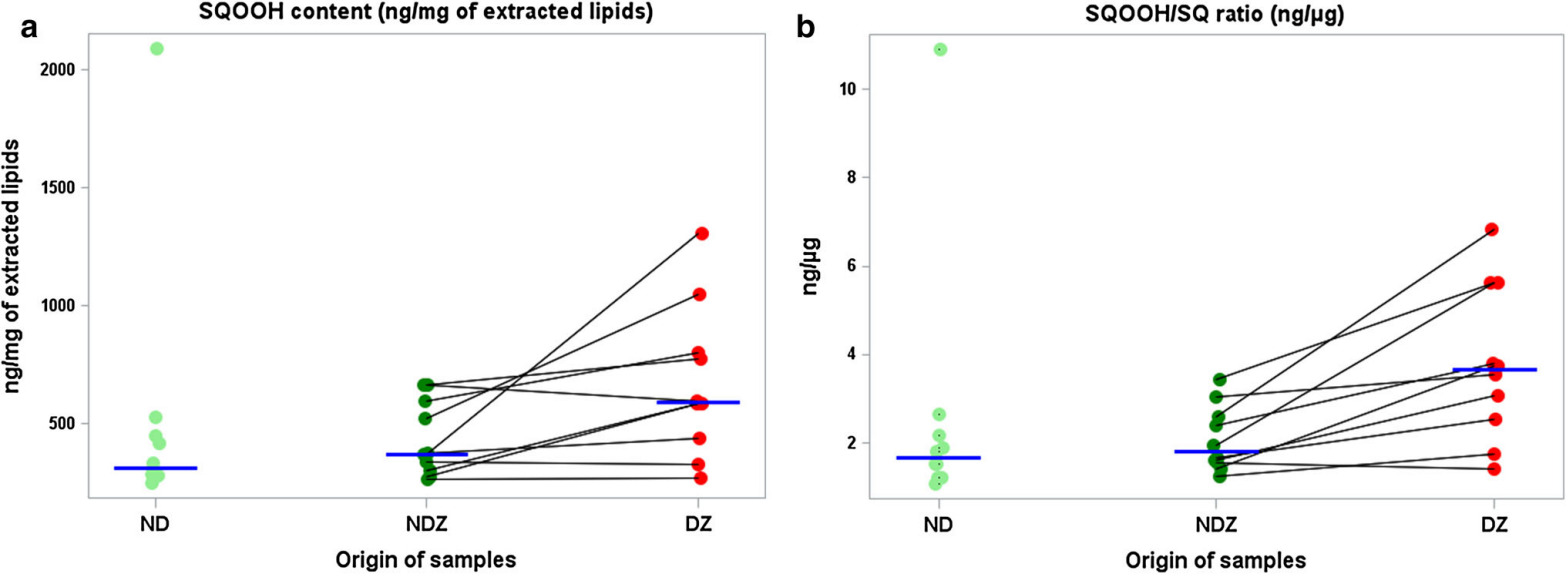
**a** Squalene monohydroperoxide (SQOOH) is increased in dandruff-affected individuals from the exploratory cohort. SQOOH (ng/mg of lipids extracted) increased significantly for individuals with dandruff. *ND* non-dandruff subjects, *NDZ* non-dandruff zone of dandruff subjects, *DZ* dandruff zone of the same dandruff subjects. Each *point* represents an individual measurement. *Blue horizontal lines* represent medians for each scalp site. Values between the two sites for each dandruff subject are linked with *black lines*. **b** The squalene monohydroperoxide/squalene (SQOOH/SQ) ratio is increased in dandruff-affected individuals in the exploratory cohort. SQOOH/SQ ratio expressed in ng/µg is shown to augment from non-dandruff subjects (ND) to the non-dandruff zones of dandruff-affected subjects (NDZ), and increased again for dandruff zones from affected patients (DZ). Each *point* represents an individual measurement, *blue horizontal lines* represent the median at each scalp zone. Values between the two sites for each dandruff subject are linked with *black lines*

Neutral lipid chromatographic profiles of both scalp zones from a given dandruff subject are illustrated by Fig. 2a. Upon chromatographic analysis, DZ of dandruff subjects showed lower levels of waxes and glycerides (as indicated by bound fatty acids) as well as squalene (Table 3) as compared to ND subjects. Squalene was not significantly different between NDZ of dandruff subjects and ND subjects, but was significantly decreased in DZ versus NDZ of the dandruff subjects (*p* < 0.05). To elucidate if a relationship exists between increased scalp oxidative stress and dandruff, we deepened our biochemical exploration into (1) the peroxidation state of lipids, using squalene monohydroperoxide (squalene oxidation product) and MDA as preliminary and final peroxidation biomarkers, respectively, and (2) enzymatic and non-enzymatic antioxidant pathways. Surprisingly we observed that squalene peroxide was not only significantly increased (+91 %) at the DZ compared to ND scalps (*p* < 0.05), but was also increased (+60 %) at the DZ compared to NDZ of the same individual (*p* < 0.05; Figs. 2b, 3a). Differences were even more striking for the squalene peroxide/squalene ratio. Indeed, dandruff subjects displayed higher levels (+100 %) at DZ when compared to NDZ (*p* < 0.01), and when compared with the ND subjects (+111 %; *p* < 0.02; Fig. 3b). MDA was significantly increased (+32 %) at DZ compared to NDZ of dandruff individuals (*p* < 0.02). Vitamin E was also increased (+21 %; *p* < 0.05). For MDA and vitamin E, intra-individual differences for specific dandruff subjects are illustrated in Online Resource 3.

**Table 3:** Biochemical characterization of the exploratory cohort of 10 control and 10 dandruff-affected individuals

	Control cohort	Dandruff-affected cohort		Statistical analysis (*p*)		
				Between groups		Intra-individual
	Non-dandruff (ND)	Non-dandruff zone (NDZ)	Dandruff zone (DZ)	ND versus NDZ	ND versus DZ	NDZ versus DZ
Cholesterol (µg/mg lipids)	9.6 (7.7–12.2)	8.6 (6.5–12.3)	9.2 (7.5–14.1)	0.65	0.94	0.23
Squalene (µg/mg lipids)	203.3 (186.9–227.2)	214.1 (193.3–218.6)	170.4 (156.4–211.1)	0.82	0.08	**<0.05**
SQOOH (ng/mg lipids)	309.7 (274.3–451.6)	371.5 (302.0–598.0)	591.0 (440.0– 800.0)	0.41	**<0.05**	**<0.05**
Ratio SQOOH/SQ (ng/µg)	1.7 (1.2–2.2)	1.8 (1.5–2.6)	3.6 (2.5–5.6)	0.36	**<0.02**	**<0.01**
MDA (ng/mg proteins)	7.5 (6.2–9.1)	7.4 (6.2–8.2)	9.8 (8.7–13.2)	0.65	0.07	**<0.02**
Catalase (UI/mg proteins)	2.9 (2.6–4.1)	2.9 (2.0–6.4)	3.7 (2.4–4.4)	0.88	0.76	0.23
Vit. E (ng/mg proteins)	53.1 (43.2–96.4)	55.1 (33.7–73.9)	66.7 (44.4–92.1)	0.82	0.60	**<0.05**
Total free fatty acids (µg/mg lipids)	376.7 (345.1–447.5)	477.7 (378.5–526.3)	419.2 (339.8–479.6)	0.13	0.60	0.49
Total bound fatty acids (µg/mg lipids)	206.3 (170.0–301.6)	141.8 (125.3–185.1)	178.2 (129.1–235.5)	0.06	0.50	**0.05**
Ratio bound/free fatty acids (%)	53.5 (34.7–67.4)	32.3 (23.8–47.0)	39.3 (21.6–87.4)	0.06	0.45	**<0.01**
Ratio free C16:0/C16:1∆6	2.9 (2.7–3.5)	3.0 (2.8–3.2)	3.3 (2.9–3.4)	0.71	0.26	0.08
Ratio bound C16:0/C16:1∆6	2.2 (1.9–2.6)	2.1 (1.7–2.5)	2.3 (2.0–3.5)	0.45	0.55	0.08
Ratio free C18:0/C18:1∆9	9.5 (8.2–12.5)	6.8 (6.6–8.2)	6.6 (5.2–7.9)	0.06	**0.02**	0.19
Ratio bound C18:0/C18:1∆9	11.6 (10.1–13.2)	10.0 (9.0–11.8)	9.5 (7.2–9.9)	0.13	**0.02**	0.63

The amounts of each lipid are expressed in relation to the total amount of extracted lipids. MDA, catalase and vitamin E amounts are expressed in relation to the total amount of collected proteins. Ratios are either without units or expressed as percentages. Values described are medians (interquartile range), with *p* values which are only exact for comparisons between groups. Significant *p* values are bolded

*C16:0* palmitic acid, *C16:1*Δ*6* sapienic acid, *C18:0* stearic acid, and *C18:1*Δ*9* oleic acid

All results concerning free or bound fatty acids are displayed in Online Resource 2 (Tables 2, 3). Detailed fatty acid analysis is explained in Online Resource 2 and illustrated in Online Resource 3 (Fig. 3). The most compelling data resulting from fatty acid analysis were the ratios of bound versus free fatty acids, as well as ratios of C16:0/C16:1, and C18:0/C18:1 (palmitic/sapienic acids and stearic/oleic acids, respectively, Table 3). The lowest ratio of bound versus free fatty acids was observed at NDZ compared to DZ of dandruff-afflicted subjects [32.3 % (23.8–47.0) vs 39.3 % (21.6–87.4), respectively, *p* < 0.01]. Free palmitic/sapienic acids tended to increase in DZ compared to NDZ [3.3 (2.9–3.4) vs 3.0 (2.8–3.2), *p* = 0.08], and the same trend was also seen for bound forms. Inversely, both the ratios of free and bound forms of stearic/oleic acids were higher in non-dandruff subjects when compared to either DZ from dandruff patients or healthy zones from control subjects, reaching significance for both free and bound forms when DZs were compared to non-dandruff individuals (*p* < 0.05 and *p* < 0.02, respectively). Quite unexpectedly, very few differences were observed between zones for the free or bound fatty acids (Online Resource 2, Tables 2, 3). For free fatty acids, differences were observed for only four fairly low abundance species: oleic (C18:1 Δ9), tridecenoic (C13:1), tetradecenoic (C14:1) and 13-methylpentadecanoic (C15:1, branched) acids. Furthermore, we did not observe any differences in the saturated/unsaturated free fatty acid ratio relating to dandruff (Online Resource 2, Table 2).

### Second validation cohort

To confirm several novel results observed within the first cohort of both dandruff-afflicted and dandruff-free subjects, a second volunteer cohort of 24 dandruff-affected volunteers was recruited and assessed.

The total amount of extracted lipids did not differ between DZ and NDZ and reached approximately 1 mg lipids/sample on both zones. We observed a significant increase in the total amount of proteins harvested from DZ as opposed to NDZ, with 523.9 (408.4–571.5) vs 463.2 (359.9–576.3) µg/ml proteins, respectively (*p* < 0.05; Table 4).

**Table 4:** Parameters assessed in the validation cohort of 24 dandruff-affected individuals

	Non-dandruff zone (NDZ)	Dandruff zone (DZ)	Intra-individual statistical *p*
Total proteins (µg/ml)	463.2 (359.9–576.3)	523.9 (408.4–571.5)	**<0.05**
Vitamin E (ng/mg proteins)	14.7 (5.1–24.1)	11.2 (2.1–20.8)	**<0.05**
Total lipids (µg)	1100 (812–1535)	1020 (775–1492)	0.15
Cholesterol (µg/mg lipids)	9.8 (6.7–13.1)	12.5 (8.1–20.8)	0.14
Squalene (µg/mg lipids)	217.4 (180.8–233.3)	164.6 (118.8–183.4)	**<0.01**
SQOOH (ng/mg lipids)	138.5 (96.1–170.7)	175.6 (97.6–232.3)	**<0.02**
Ratio SQOOH/SQ (ng/µg)	0.70 (0.40–0.94)	1.0 (0.67–1.62)	**<0.01**

Values described are medians (interquartile range) with *p* values, significant *p* values are in bold

**Fig. 4:**
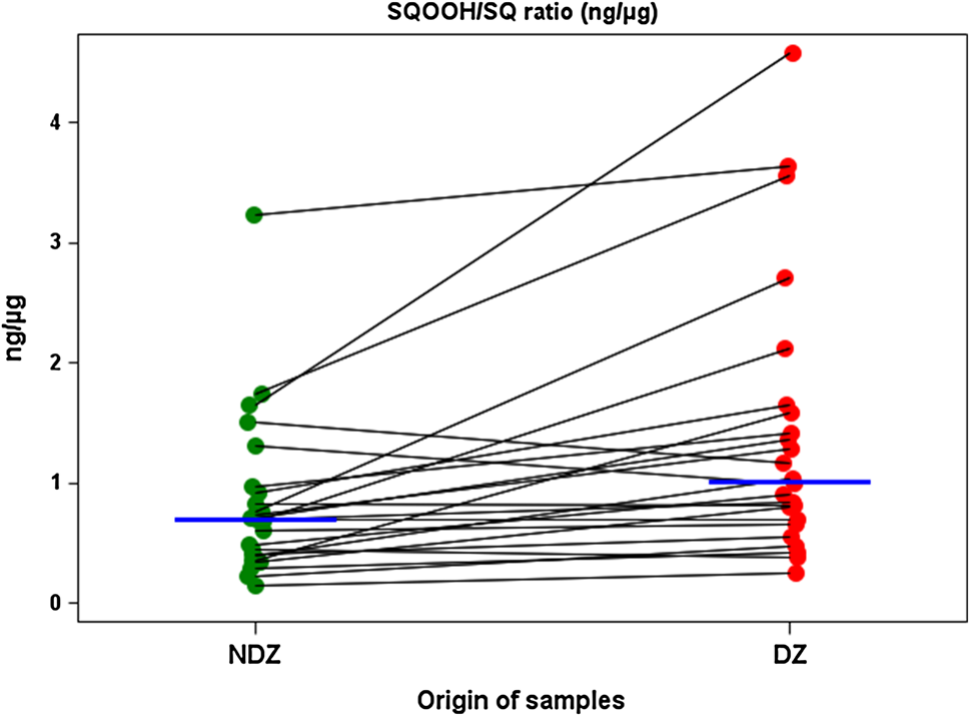
The squalene monohydroperoxide/squalene (SQOOH/SQ) ratio was increased on dandruff-affected zones of the validation cohort. SQOOH/SQ ratio expressed in ng/µg was shown to augment from non-dandruff zones (NDZ) to dandruff zones (DZ) for the 24 affected subjects. Each *point* represents an individual measurement, *blue horizontal lines* represent the median at each scalp zone. Values between the two sites for each subject are linked with *black lines*

As was clearly observed in the exploratory cohort, this validation cohort displayed decreased amounts of squalene, with a corresponding increase in squalene peroxide in DZ compared to NDZ (*p* < 0.01 and *p* < 0.02, respectively), resulting in a 43 % increase in SQOOH/SQ ratio (*p* < 0.0001; Fig. 4). In the first cohort, we observed a significant increase in intra-individual vitamin E levels (*p* < 0.05) in DZ compared to NDZ, which surprisingly was inversed in the second cohort (*p* < 0.05).

## Discussion

Though biochemical and lipid profiles of the skin have been thoroughly characterized, only few studies have dealt with scalp. We thus aimed to perform a large biochemical description of lipids collected from dandruff and dandruff-free scalps, with an entirely novel sampling procedure. Using the wealth of generated data, our secondary objective was to shed more light on dandruff etiological pathways.

To validate our novel sampling methods and analytical techniques, we first quantified forehead sebum in healthy volunteers (Online Resource 2, Table 4), and found similar sebum component proportions as described in the literature [20], indicating sound sampling and analytical methods for conducting biochemical exploration of skin surface lipids. On these six healthy subjects, we also compared forehead and scalp surfaces in terms of lipid composition. With much higher levels of free fatty acids and fewer glycerides, scalp lipid composition was unique compared to the facial skin (Online Resource 2, Table 4). This difference is perhaps due to a greater abundance of colonizing lipophilic microorganisms (*M. restricta*, *P. acnes*) characterized by strong lipase activity.

From the exploratory study cohort, approximately 4 mg lipid samples were collected, whereas only 1 mg lipid samples were collected from the validation cohort. This variation could be explained by several factors. The exploratory cohort was composed of 19 men and 1 woman, whereas the second had a more equal male:female ratio. In actual fact, we noted that the average amount of lipids collected from the female validation cohort subjects (whether dandruff or non-dandruff zones), was less than that collected from the males (979 vs 1291 mg, respectively). This result correlates with several studies, one of which was performed on 713 Chinese subjects. This study demonstrated that forehead zones close to the hairline had higher sebum content in males aged 13–70 years, compared to age-matched females [21]. Our observed differences could equally be explained by seasonal changes. The first exploratory study was performed in Paris during the late summer/early autumnal months of September and October, whereas the validation study took place in Lyon during early spring (March/April). Weather was thus much warmer during the exploratory study, which could explain the greater quantity of sebum collected. It has been shown that for every one degree increase in ambient air temperature, sebum excretion increases at least 10 %, even though the number of active sebaceous glands remains stable [35]. We also cannot exclude a difference between the two experimenters responsible for sampling each cohort. In any case, the fact that no difference was observed in the total amounts of sampled lipids between the three zones from the exploratory cohort, and the two zones in the validation cohort, confirms the relevance of using this type of sampling method. In the second cohort, we observed a significant increase in the total amount of proteins harvested from DZ as opposed to NDZ, which was not observed for the first cohort (Table 4). This may be due to higher global dandruff scores in the second cohort (6.7 ± 1.4) than in the first (5.5 ± 0.7), indicating a more abnormal scalp stratum corneum which could favor surface protein collection.

Of all the biochemical differences observed between the different scalp zones, the most compelling was the dramatically increased level of SQOOH squalene peroxide at dandruff zones observed in both cohorts. Squalene is an important sebum component [28] only found on the skin of humans and certain other water mammals [19]. Squalene is thought to act as a lipid sensor for solar UV exposure, and as a photoprotective barrier for the hairless human skin against such oxidative stress, acting as a sacrificial target when other defense mechanisms are exhausted [10, 19, 25, 27, 40]. However, in the hair-covered scalp microenvironment, the effects of squalene, and its breakdown product, SQOOH, are completely unknown. Other studies have demonstrated that SQOOH plays a key role in the development of facial acne, by stimulating keratinocyte proliferation and inflammation [4, 28, 29, 40], and could also be a relevant biomarker for the effects of atmospheric pollution upon the skin [33]. In addition, SQOOH can penetrate cell layers and influence the immune system [2, 10]. As dandruff is characterized by inflammation and keratinocyte hyperproliferation, the combination of all these elements suggests that SQOOH could play a role in the development of dandruff. Our conviction also arises from as-yet unpublished clinical data indicating that increased squalene peroxidation occurs before dandruff becomes clinically visible. UV exposure induces squalene peroxidation on hairless human skin [25], but only slightly reaches the hair-covered scalp surface [8]. In addition, in our experience, dandruff incidence tends to be higher in winter than in summer. Therefore, it is unlikely that UV exposure is a major cause of increased squalene peroxidation observed with dandruff. Alternatively, as *Malassezia* can induce squalene peroxidation in vitro [9], we can hypothesize that this SQOOH increase is at least partially due/linked to increased *M. restricta* prevalence on dandruff scalps [6, 42].

Catalase and MDA oxidative stress markers are both increased in SD scalps [30], therefore we further investigated oxidative stress pathways in both dandruff and non-dandruff subjects. Hydrogen peroxide accumulation can result in cellular damage by oxidizing proteins, DNA, and lipids. Catalase is ubiquitously expressed in nearly all living cells exposed to oxygen, and alleviates oxidative damage by catalyzing hydrogen peroxide. On the scalp surface, catalase activity could be explained by its abundant colonization by catalase-positive bacteria such as *Propionibacterium acnes* and *Staphylococcus epidermidis* [6]. However, catalase activity was not altered between any of the selected scalp zones. Conversely, MDA was increased at DZ versus NDZ from affected volunteers. Vitamin E is also a known antioxidant due to its ability to inhibit lipid peroxidation by trapping hydroperoxide radicals [5]. The vitamin E results from both studies differed in two aspects: the total quantities and the direction of trends at both dandruff and non-dandruff zones. In the first exploratory cohort, we collected approximately 60 ng of vitamin E per mg of total protein, but only 12 ng in the validation cohort. This significant difference is probably linked to the differences in amounts of collected samples between the two cohorts. Indeed, there seems to be a strong link between the quantity of vitamin E and the amount of excreted sebum, as has been shown previously by other authors [34]. We observed both reduced amounts of vitamin E, and fewer lipids collected from the validation cohort as compared to the exploratory cohort, and with similarly reduced ratios; 80 % less vitamin E, and 75 % fewer total lipids. This result supports our conviction that the collection and quantification of vitamin E were undertaken under optimal conditions. In contrast, we are unable to find an explanation for the reduced levels of vitamin E at non-dandruff zones compared to dandruff zones in the exploratory cohort, whereas the exact inverse was observed in the validation cohort. Whether this conflicting result is due to differences in dandruff severity between both cohorts, individual skin responses to oxidative attack, the action of the scalp microbiome, or another as-yet unidentified third player, is currently unknown. More analysis must be performed on samples harvested from larger panels of dandruff subjects under different conditions such as season to better establish the possible links between vitamin E and dandruff. Together, these results seem to indicate that dandruff-afflicted scalps are undergoing oxidative stress, but whether this is a root cause or solely an effect remains to be established.

The biochemical profiles of an extensive range of free and bound fatty acids were also explored. Few differences were observed between zones for the free or bound fatty acids, even given the large number of assessed biomarkers. These relatively small differences observed are difficult to interpret because they generally concerned scarce fatty acids and the results did not form a continuum between dandruff-free subjects, NDZ and DZ of dandruff subjects, as was seen for SQOOH. Surprisingly, the lowest ratios of bound to free fatty acids were observed at non-dandruff sites of affected individuals, suggestive of higher levels of lipase activity. Therefore, it appears that dandruff is not strongly linked to free fatty acid release. This result could seem counterintuitive as dandruff is often described as associated with increased numbers of *Malassezia*, known for their lipase activity. However, *P. acnes*, with a correspondingly high lipase activity, is also present in large numbers on normal scalps [6, 42], and could account for these observations. Taken together, we can hypothesize that increased free fatty acids and decreased glycerides are consequences of intense lipase activity from the scalp microbiome, consistently abundant in lipophilic species such as *Malassezia restricta* and *P. acnes*, whatever the dandruff status [6, 42].

Free and bound stearic/oleic acid ratios were higher in non-dandruff subjects indicating an increase in the production of oleic acid at the expense of stearic acid at dandruff scalp zones [45]. These results neither confirm nor refute the previous studies demonstrating that oleic acid can induce dandruff when applied to seemingly normal-looking scalps of dandruff subjects after anti-dandruff treatment, whereas it is without effect on non-dandruff subjects. Although free oleic acid is reduced in non-dandruff subjects, oleic acid levels are identical at both ‘normal’ and affected zones of dandruff subjects, despite their clinical differences. Both free and bound palmitic/sapienic acid ratios tended to increase in dandruff zones when compared to non-dandruff zones of affected individuals. This suggests a constitutive deficit in sapienic production, with more abundant palmitic acid in dandruff-affected areas [12, 29, 34]. As sapienic acid is a known antimicrobial fatty acid [3], reduced levels of this acid could provoke the disequilibrium in microbial populations recently reported on the scalps of dandruff-affected individuals [6]. Furthermore, we did not observe any dandruff-related difference in the ratio of saturated/unsaturated free fatty acids. This result argues against the hypothesis that in vivo, after hydrolyzing sebaceous triacylglycerol components, *Malassezia* specifically utilizes saturated fatty acids to support its growth, while leaving behind unsaturated fatty acid waste products which irritate the skin and promote scalp flaking in susceptible individuals [11, 37].

In summary, results from this unparalleled lipid profile characterization of both healthy and dandruff-affected scalps showed a surprisingly large increase in levels of SQOOH. In combination with increased MDA, these results suggest an emerging trend of amplified oxidative stress in dandruff-affected scalps, similar to that observed for acne-affected skin and for scalp seborrheic dermatitis [29, 30]. This is the very first evidence that SQOOH may play a role in the development of dandruff. Based on our results and the ability of *Malassezia* to induce squalene peroxidation [9], we propose the following dandruff model: increased levels of *Malassezia* result in increased SQOOH, which in turn penetrates the stratum corneum and stimulates inflammation and keratinocyte proliferation, generating characteristic clinical dandruff signs, such as altered skin barrier function, flakes and itching. This scalp condition could in turn favor increased *Malassezia* growth, and maintain dandruff status. To explore this hypothesis, the next step would be to develop an in vitro assay to determine whether *M. restricta* is really able to induce squalene peroxidation, and then to identify compounds preventing this process. Model validation would be achieved by demonstrating that such compounds effectively reduce squalene peroxidation in vivo, and, in parallel, exert an anti-dandruff effect.

## Electronic supplementary material

Supplementary material 1 (PDF 88 kb)

Supplementary material 2 (PDF 84 kb)

Supplementary material 3 (PDF 116 kb)
