# Supplementary material for: Exploration of scalp surface lipids reveals squalene peroxide as a potential actor in dandruff condition
Source: Arch Dermatol Res. 2016 Feb 3;308:153–63. doi: 10.1007/s00403-016-1623-1 (PMC4796319; doi:10.1007/s00403-016-1623-1)
Supplement: Supplementary file 1 — Supplementary material 1 (PDF 88 kb) [file 403_2016_1623_MOESM1_ESM.pdf]

Article Title: Exploration of scalp surface lipids reveals squalene peroxide as a potential actor in dandruff condition

Journal: Archives of Dermatological Research

Authors: Roland Jourdain, Alain Moga, Philippe Vingler, Charles el Rawadi, Florence Pouradier, Luc Souverain, Philippe Bastien, Nicolas Amalric, Lionel Breton

Corresponding Author: Roland Jourdain - L'OREAL Research & Innovation, Aulnay-sous-Bois, France - Email: rjourdain@rd.loreal.com

Supplementary Information

Additional ethical considerations

To comply with ethical requirements, subjects from both studies (L'OREAL: ACR/SYL1/1130, Intertek Paris: 184-HAI-DAN-11-05; L'OREAL: ACR/SPAD/1123, DERMSCAN Lyon: 11E1945), received verbal and written information concerning the study. This information explained the nature, purpose and risks of the study and emphasized that study participation was voluntary, and that the subject might withdraw from the study at any time, for any reason. All subjects had the opportunity to ask study-related questions and were given sufficient time to consider their participation before consenting. Written informed consent was obtained prior to any study-related procedure being carried out. All data were analyzed anonymously and steps were taken to protect the identities of all participants. According to the following French national laws; "*Informatique et liberté*" law from January 6th 1978, modified by-law No. 94–548 of July 1st 1994, and law 2004–801 of August 6th 2004, both subject databases were declared to the ''*Commission Nationale de l'Informatique et des Libertés*'' (National Data Processing and Freedom Commission)''.

Further subject selection criteria

Any female subjects were not pregnant and used appropriate contraception during the study period. Subjects were excluded if they did not meet the above inclusion criteria, or if they had the following; they had any systemic disorder or skin disease which might significantly affect interpretation of the study results, e.g. eczema, psoriasis; if they were taking medication which may affect the scientific validity of the study, or the subject's wellbeing; if there was a history of, or planned medical/surgical events; suffering from acute or chronic disease; a history of allergies; pregnancy or lactation; receiving isotretinoin treatment (Curcné®, Procuta®; etc); having used a topical scalp anti-dandruff treatment in the two weeks prior to the first assessment visit; having used an oral scalp anti-dandruff treatment in the three months prior to the first assessment visit; having had cosmetic or medical anti-hair loss treatment in the three or six months prior to the first assessment visit; displaying alopecia of a Hamilton degree >5a at the crown; subjects with frizzy and/or white/gray and/or very short hair (< 2 cm long); excessive or intensive exposure to sunlight (natural or artificial) prior to or during the study; subjects who declared to have been deprived of their freedom by administrative or legal decision, or who are under guardianship; subjects who cannot be contacted by telephone in case of emergency, clinical center employees, within an exclusion period, or participating in another biomedical research study. An interval of between two to three weeks was observed between the first assessment visit, and the second sampling visit, during which time, all participants replaced their normal shampoo with the neutral study shampoo stored at room temperature. During the study, the participants used the neutral study shampoo twice a week, avoided using oral or topical hair and scalp treatments for dandruff or hair loss; did not cut, perm, straighten, color or bleach the hair;

avoided using cosmetic hair products; avoided swimming, attending saunas, spas or hammam; avoided exposure to ultraviolet rays, either natural or artificial; avoided new medications (excepting paracetamol), and resisting scalp scratching. At the second visit, participants returned any unused shampoo along with a study diary. Prior to sampling, participants acclimatized in the test room for 20 minutes, without consuming food or hot drinks.

Additional information on sample collection and biochemical exploration

Lipid species analysis

When screening for neutral lipids such as free fatty acids, squalene, cholesterol, bound forms such as waxes, cholesterol esters and glycerides, external calibration was performed for the lipid families of interest, using fresh references for each analysis to avoid breakdown products. Neutral lipids were extracted from the sebum homogenate according to the Bligh Dyer (liquid/liquid extraction) method and then completely dried under nitrogen at 30°C. Residues were resuspended in 50µl of dichloromethane. For spectrometry analysis, an Agilent 6890N (Santa Clara, CA, USA) gas chromatography unit equipped with an on-column injector was coupled to a Agilent 5975 Mass Selective Detector mass spectrometer via a high-temperature interface. Separation was achieved using a 30m × 0.25mm × 0.1µm ZB-5HT capillary column (Phenomenex, Torrance, CA). Helium was used as a carrier gas at a constant flow of 1ml/min. Injector and transfer line temperatures were set to 300°C and 380°C, respectively. Oven temperatures were programed as follows; a 50°C hold for 30sec, heating to 250°C at a rate of 20°C/min; increased to 300°C at 35°C/min; a 5 minute hold, increased to 380°C at 35°C/min, with a final hold at 380°C for 15min. EI mass spectra were recorded in a total ion current (TIC) monitoring mode. EI-MS operating conditions were source temperature at 250°C, ionizing energy at 70 eV, and a scan range from 50 to 1000m/z.

As for the neutral lipids, free fatty acids were extracted from the sebum homogenate according to the Bligh Dyer method. FAMEs (methylated fatty acids) were obtained via extraction into an organic hexane phase and dried under nitrogen at 60°C. Dried residues were resuspended in 100µl of hexane. Gas chromatography separation and mass spectrometry was achieved using a 60m × 0.25mm × 0.25µm VF-23MS capillary column (Agilent, Santa Clara, CA, USA). The VF-23ms column features a unique combination of high polarity and low bleed, which enables more accurate fatty acid analysis and the ability to separate C16 and C18 fatty acid isomers. Fatty acid separation was assessed using our in-house reference substances. Fatty acid identification was obtained by comparing retention times against those of reference substances, and via mass spectrophotometry. As illustrated by the Supplementary figure 3, palmitoleic acid (C16:1 Δ9) and sapienic acid (C16:1 Δ6) were separated simultaneously from palmitic acid (C16:0) during the same VF-23ms run with the ramp temperature described below. Helium was used as a carrier gas at a constant flow rate of 1ml/min. Both injector and transfer line temperatures were set to 250°C. Oven temperatures were programmed to hold at 100°C for 2min, to increase to 140°C at 40°C/min; then to 152°C at 0.25°C/min; to 250°C at 40°C/min, with a final hold at 40°C for 10min. EI mass spectra were recorded in total ion current (TIC) monitoring mode. The operating conditions for EI-MS were source temperature at 250°C, ionizing energy at 70eV, and a scan range from 50 to 1000m/z.

Bound fatty acids were obtained from the waxes, cholesterol esters and glycerides in the sebum homogenate using proprietary dual liquid/solid extraction methods. Bound fatty acids were then further purified by saponifying and extracting the primitive lipid forms using the Bligh Dyer procedure. Fatty acids were then methylated, dried, resuspended and chromatography performed as for the free fatty acids above.

Squalene monohydroperoxide (SQOOH) analysis

Samples were extracted from the sebum homogenate using a proprietary double liquid/liquid extraction method, evaporated under nitrogen at 60°C, and the residue resuspended in 100µl of methanol. As no commercially available squalene monohydroperoxide standards were available for chromatographic analysis, an in-house standard was synthesized using a procedure based on squalene photo-oxygenation in the presence of a photosensitizer (Bengal rose) [13,25]. Different molecules were formed using this process: mono-, di-, and triperoxides, as well as epoxides and endoperoxides. For this reason, semi-preparative liquid chromatography was used. The synthetic squalene peroxide substance had several positional isomers, so proprietary purification was performed to obtain the 11-SQOOH isoform where the –OOH group is located at position 11 on the SQOOH backbone.

An UltiMate 3000 (Dionex, Sunnyvale, CA, USA) liquid chromatography system coupled to a single stage MSQ Plus detector (Fisher Scientific, Waltman, MA, USA) was used for the detection of SQOOH. In this LC/MSD system, two mobile phases [M1, water] and [M2, acetonitrile] were eluted at a flow rate of 0.2ml/min. The mobile phases were programed consecutively as follows; an isocratic elution of 50% M1 from 0 to 15min, a linear gradient of 50–0% M1 between 15 and 20min, an isocratic elution of 0% M1 for 45min, and an isocratic elution of 50% M1 from 45.1 to 60min for column equilibration. Injection volume was 20µl, and column temperature was maintained at 40°C. For MS detection, atmospheric pressure chemical ionization was used as the ion source, the probe temperature was set at 500°C, with a cone of 50. Positive ion spectra were recorded in the range 50–450m/z [24].

Malondialdehyde (MDA) analysis

Swab homogenates were centrifuged at 20 000 *g* for 5min for all subsequent analyses. Acid hydrolysis and PFB derivation was performed, followed by liquid/liquid extraction, with the organic phase dried under nitrogen at 60°C. Residues were resuspended in 50µl of hexane. Gas spectrometry and separation was achieved using a 30m × 0.25mm × 0.10µm ZB-5HT capillary column, and helium was used as a carrier gas at a constant flow of 1ml/min. Both injector and transfer line temperatures were set to 250°C. Pulsed splitless mode (25 psi pulse) was used. Oven temperatures started with a 30sec hold at 50°C, increased to 180°C at 25°C/min; then to 250°C at 5°C/min; to 300°C at 25°C/min, with a final hold for 10min. Negative ion chemical ionization with methane reagent gas was performed using Selected Ion Monitoring (SIM).

Analysis of the enzymatic detoxification system

Biochemical markers were analyzed from 20 µl swab samples. Catalase activity was determined using the Catalase Fluorometric Detection kit (Enzo Life Sciences, Farmingdale, NY, USA).

Analyzing the non-enzymatic detoxification system (Vitamin E)

Swab sample homogenates were were hydrolyzed, washed with an organic solvent (ethanol) , and residue resuspended in 100 µl of methanol. The UltiMate 3000 liquid chromatography system coupled to a FLD-3000 fluorescence detector (Dionex, Sunnyvale, CA, USA) was used. The two mobile phases [M1, water] and [M2, methanol] were eluted at a flow rate of 0.5ml/min. The mobile phases were programed consecutively as follows; an isocratic elution of 50% M1 from 0 to 5min, a linear gradient of 50–10% M1 between 5 and 20min, an isocratic elution of 10% M1 for 15min, and an isocratic elution of 50% M1 from 35.1 to 45min for column equilibration. Injection volume was 20µl and column temperature was maintained at 30°C. The excitation/emission wavelengths were respectively set to 295 and 340nm.
