# Supplementary material for: Exploration of scalp surface lipids reveals squalene peroxide as a potential actor in dandruff condition
Source: Arch Dermatol Res. 2016 Feb 3;308:153–63. doi: 10.1007/s00403-016-1623-1 (PMC4796319; doi:10.1007/s00403-016-1623-1)
Supplement: Supplementary file 2 — Supplementary material 2 (PDF 84 kb) [file 403_2016_1623_MOESM2_ESM.pdf]

Article Title: Exploration of scalp surface lipids reveals squalene peroxide as a potential actor in dandruff condition

Journal: Archives of Dermatological Research

Authors: Roland Jourdain, Alain Moga, Philippe Vingler, Charles el Rawadi, Florence Pouradier, Luc Souverain, Philippe Bastien, Nicolas Amalric, Lionel Breton

Corresponding Author: Roland Jourdain - L'OREAL Research & Innovation, Aulnay-sous-Bois, France - Email: rjourdain@rd.loreal.com

Supplementary Tables

Supplementary Table 1 Scalp surface biomarkers that were quantitatively assessed

Biomarkers	Common name
Catalase	NA
MDA	Malondialdehyde
Vitamin E	NA
Cholesterol	NA
Squalene	NA
SQOOH	Squalene monohydroperoxide
C10:0	Decanoic acid
C12:0	Lauric acid
C12:0, branched	11-Methyldodecanoic acid
C13:0, branched	12-Methyltridecanoic acid
C13:1	Tridecenoic acid
C14:0	Myristic acid
C14:0, branched	13-Methyltetradecanoic acid
C14:1	Tetradecenoic acid
C14:1, branched	12-Methyltetradecanoic acid
C15:0	Pentadecanoic acid
C15:0, branched	14-Methylpentadecanoic acid
C15:1	Pentadecenoic acid
C15:1, branched	13-Methylpentadecanoic acid
C16:0	Palmitic acid
C16:1 Δ6	Sapienic acid
C16:1 Δ9	Palmitoleic acid
C17:0	Heptadecanoic acid
C17:1	Heptadecenoic acid
C18:0	Stearic acid
C18:1 Δ8	8-Octadecenoic acid
C18:1 Δ9	Oleic acid
C18:1 Δ11	11-Octadecenoic acid
C18:2 Δ5,8	Sebaleic acid
C18:2 Δ9,12	Linoleic acid
C20:0	Arachidic acid
C20:1	Eicosenoic acid
C22:0	Behenic Acid

Supplementary Table 2 Biochemical characterization of the cohort of 10 control and 10 dandruff-affected volunteers – Free fatty acids (in decreasing order of quantity)

	Control cohort	Dandruff-affected cohort		Statistical analysis (p)		
				Between groups		Intra-individual
	Non-dandruff (ND)	Non-dandruff zone (NDZ)	Dandruff zone (DZ)	ND *vs* NDZ	ND *vs* DZ	NDZ *vs* DZ
C16:0 (µg/mg)	149.5 *(137.4 – 168.9)*	190.1 *(152.2 – 215.2)*	166.5 *(126.3 – 199.4)*	0.17	0.50	0.56
C16:1 Δ6 (µg/mg)	52.2 *(40.5 – 65.7)*	65.1 *(53.0 – 71.7)*	52.3 *(37.3 – 71.6)*	0.41	0.94	0.28
C14:0 (µg/mg)	31.8 *(28.5 – 38.2)*	41.7 *(37.1 – 46.1)*	38.4 *(29.1 – 40.8)*	0.15	0.65	0.06
C15:0 (µg/mg)	32.0 *(26.5 – 37.0)*	36.6 *(28.5 – 41.4)*	31.1 *(28.4 – 40.0)*	0.41	1.00	0.70
C18:1 Δ8 (µg/mg)	22.8 *(18.2 – 29.3)*	31.7 *(19.5 – 35.5)*	24.3 *(15.6 – 36.5)*	0.23	0.65	0.11
C18:0 (µg/mg)	21.9 *(20.4 – 26.0)*	25.8 *(24.9 – 28.3)*	25.7 *(21.4 – 27.0)*	0.13	0.33	0.49
C17:0 (µg/mg)	7.4 *(5.9 – 10.1)*	9.9 *(7.2 – 13.6)*	8.5 *(7.4 – 12.3)*	0.23	0.45	0.43
C18:2 Δ5,8 (µg/mg)	7.7 *(5.5 – 8.7)*	7.9 *(6.0 – 9.0)*	8.3 *(5.7 – 10.7)*	0.65	0.60	0.85
C17:1 (µg/mg)	6.7 *(4.8 – 8.7)*	7.5 *(5.5 – 8.1)*	6.2 *(3.3 – 10.8)*	0.71	0.88	0.56
C14:1, branched (µg/mg)	5.1 *(4.1 – 6.9)*	7.1 *(6.5 – 7.6)*	5.9 *(5.4 – 6.5)*	0.15	0.41	0.11
C18:2 Δ9,12 (µg/mg)	4.7 *(3.9 – 7.5)*	5.5 *(4.3 – 6.1)*	5.4 *(3.8 – 10.6)*	0.55	0.50	0.85
C22:0 (µg/mg)	4.1 *(3.7 – 7.1)*	4.4 *(3.2 – 5.3)*	4.2 *(3.1 – 9.9)*	0.76	0.88	0.56
C15:1 (µg/mg)	4.0 *(2.0 – 5.1)*	4.0 *(3.5 – 4.3)*	3.3 *(2.8 – 4.5)*	0.88	0.41	0.37
C14:1 (µg/mg)	3.9 *(3.0 – 4.7)*	4.0 *(3.8 – 5.1)*	3.6 *(3.4 – 3.9)*	0.60	0.41	**0.02**
C15:1, branched (µg/mg)	2.8 *(1.7 – 4.6)*	4.7 *(2.6 – 7.5)*	3.4 *(2.3 – 7.1)*	0.11	0.36	**0.01**
C14:0, branched (µg/mg)	2.8 *(2.1 – 3.7)*	3.1 *(2.7 – 3.9)*	2.7 *(2.6 – 3.5)*	0.45	0.76	0.07
C18:1 Δ9 (µg/mg)	2.3 *(1.7 – 3.1)*	3.9 *(3.1 – 4.3)*	3.6 *(2.7 – 5.2)*	**0.01**	**0.02**	0.92
C15:0, branched (µg/mg)	2.3 *(1.6 – 2.9)*	2.3 *(2.2 – 2.5)*	2.3 *(1.8 – 2.6)*	0.65	0.76	0.49
C13:1 (µg/mg)	1.2 *(0.8 – 4.0)*	3.5 *(0.8 – 5.4)*	2.3 *(0.8 – 2.6)*	0.26	0.55	**0.01**
C20:0 (µg/mg)	1.2 *(0.7 – 1.7)*	0.8 *(0.4 – 1.0)*	0.7 *(0.6 – 0.8)*	0.07	0.06	0.77
C12:0 (µg/mg)	0.9 *(0.9 – 1.3)*	1.0 *(0.9 – 1.2)*	1.2 *(0.8 – 1.3)*	0.50	0.54	1.00

C13:0, branched (µg/mg)	0.9 *(0.5 – 1.0)*	0.8 *(0.7 – 0.9)*	0.8 *(0.6 – 1.2)*	0.94	0.76	0.77
C12:0, branched (µg/mg)	0.8 *(0.6 – 0.9)*	0.7 *(0.7 – 0.8)*	0.8 *(0.6 – 1.0)*	0.88	0.50	0.70
C20:1 (µg/mg)	0.7 *(0.0 – 2.2)*	0.9 *(0.0 – 2.4)*	1.6 *(0.0 – 2.4)*	0.72	0.53	0.69
C16:1 Δ9 (µg/mg)	0.7 *(0.5 – 1.0)*	0.8 *(0.6 – 0.9)*	0.8 *(0.8 – 1.2)*	0.36	0.26	0.92
C18:1 Δ11 (µg/mg)	0.5 *(0.3 – 0.6)*	0.7 *(0.3 – 0.8)*	0.7 *(0.4 – 0.9)*	0.33	0.06	1.00
C10:0 (µg/mg)	0.3 *(0.0 – 0.7)*	0.0 *(0.0 – 0.2)*	0.0 *(0.0 – 0.2)*	0.10	0.19	1.00
ratio C18:0/C18:1 Δ9	9.5 *(8.2 – 12.5)*	6.8 *(6.6 – 8.2)*	6.6 *(5.2 – 7.9)*	0.06	**0.02**	0.19
ratio C16:0/C16:1 Δ6	2.9 *(2.7 – 3.5)*	3.0 *(2.8 – 3.2)*	3.3 *(2.9 – 3.4)*	0.71	0.26	0.08
ratio saturated/ unsaturated	2.3 *(2.1 – 2.5)*	2.3 *(2.2 – 2.6)*	2.3 *(2.2 – 2.5)*	0.60	0.50	0.19

Values described are medians *(interquartile ranges)*, with *p* values which are only exact for comparisons between groups. Significant *p* values are bolded. Free fatty acid ratio values are listed at the end of the table and are separated by a dashed line.

Supplementary Table 3 Biochemical characterization of the cohort of 10 control and 10 dandruff-affected volunteers – Bound fatty acids (in decreasing order of quantity)

	Control cohort	Dandruff-affected cohort		Statistical analysis (p)		
				Between groups		Intra-individual
	Non-dandruff (ND)	Non-dandruff zone (NDZ)	Dandruff zone (DZ)	ND *vs* NDZ	ND *vs* DZ	NDZ *vs* DZ
C16:0 (µg/mg)	70.4 *(50.6 – 106.4)*	45.9 *(37.0 – 56.3)*	52.0 *(45.2 – 80.0)*	0.06	0.41	0.08
C16:1 Δ6 (µg/mg)	30.5 *(21.4 – 45.8)*	20.9 *(18.6 – 21.8)*	20.7 *(18.8 – 24.2)*	0.07	0.06	0.85
C18:0 (µg/mg)	23.0 *(18.8 – 28.8)*	17.0 *(14.4 – 20.0)*	18.3 *(12.0 – 37.1)*	0.17	0.50	0.16
C18:1 Δ8 (µg/mg)	14.4 *(7.3 – 18.6)*	8.9 *(5.3 – 13.2)*	11.3 *(7.5 – 13.4)*	0.17	0.55	0.23
C15:0 (µg/mg)	11.2 *(6.0 – 14.5)*	4.9 *(4.2 – 9.5)*	6.0 *(5.3 – 6.8)*	**0.05**	0.17	0.32
C14:0 (µg/mg)	9.3 *(5.2 – 13.5)*	6.8 *(4.5 – 8.0)*	10.1 *(6.1 – 15.5)*	0.22	0.76	**0.05**
C18:2 Δ5,8 (µg/mg)	6.6 *(5.1 – 8.6)*	5.3 *(4.0 – 6.3)*	5.3 *(3.8 – 11.0)*	0.29	0.54	0.77
C18:2 Δ9,12 (µg/mg)	4.6 *(3.8 – 7.5)*	4.6 *(3.4 – 5.5)*	4.4 *(3.2 – 11.0)*	0.94	0.94	0.92
C17:1 (µg/mg)	4.4 *(2.4 – 5.5)*	2.0 *(1.8 – 3.8)*	2.3 *(1.7 – 2.6)*	**0.04**	0.07	0.92
C22:0 (µg/mg)	4.0 *(3.5 – 6.9)*	4.4 *(2.9 – 5.1)*	3.8 *(2.7 – 9.3)*	0.94	0.94	1.00
C17:0 (µg/mg)	3.8 *(1.7 – 4.6)*	2.0 *(1.6 – 3.1)*	2.5 *(2.1 – 3.0)*	0.26	0.50	**0.05**
C14:1, branched (µg/mg)	2.6 *(1.8 – 4.4)*	2.6 *(1.4 – 3.2)*	3.2 *(1.9 – 7.6)*	0.60	0.50	0.11
C15:1 (µg/mg)	2.1 *(1.6 – 2.3)*	1.3 *(1.1 – 1.6)*	1.4 *(1.3 – 1.6)*	**0.03**	**0.02**	0.77
C18:1 Δ9 (µg/mg)	1.9 *(1.6 – 2.1)*	1.7 *(1.5 – 2.0)*	1.9 *(1.4 – 4.0)*	0.55	0.33	0.08
C14:0, branched (µg/mg)	1.5 *(1.3 – 1.8)*	1.2 *(0.9 – 1.6)*	1.8 *(0.8 – 2.2)*	0.23	0.94	0.11
C14:1 (µg/mg)	1.5 *(1.3 – 1.9)*	1.3 *(0.9 – 1.6)*	1.4 *(1.2 – 1.9)*	0.20	0.50	0.32
C15:1, branched (µg/mg)	1.3 *(1.0 – 2.0)*	1.3 *(0.8 – 2.4)*	1.5 *(0.7 – 2.4)*	0.94	0.82	0.70
C15:0, branched (µg/mg)	1.3 *(1.2 – 1.6)*	0.8 *(0.6 – 1.1)*	1.0 *(0.7 – 1.6)*	**0.05**	0.15	**0.04**
C13:1 (µg/mg)	0.6 *(0.3 – 1.1)*	0.7 *(0.4 – 0.8)*	0.5 *(0.3 – 1.1)*	1.00	0.79	0.92
C16:1 Δ9 (µg/mg)	0.6 *(0.4 – 0.9)*	0.5 *(0.4 – 0.6)*	0.4 *(0.3 – 0.8)*	0.65	0.36	1.00
C13:0, branched (µg/mg)	0.5 *(0.4 – 0.9)*	0.5 *(0.3 – 0.8)*	0.5 *(0.3 – 1.3)*	0.65	0.65	0.70

C18:1 Δ11 (µg/mg)	0.3 *(0.3 – 0.5)*	0.3 *(0.2 – 0.7)*	0.4 *(0.1 – 0.8)*	0.94	0.55	0.70
C12:0, branched (µg/mg)	0.4 *(0.3 – 0.5)*	0.2 *(0.2 – 0.3)*	0.2 *(0.2 – 0.5)*	0.07	0.13	0.65
C20:0 (µg/mg)	0.3 *(0.3 – 0.6)*	0.2 *(0.1 – 0.5)*	0.3 *(0.1 – 0.4)*	0.13	0.33	0.56
C12:0 (µg/mg)	0.3 *(0.2 – 0.4)*	0.1 *(0.0 – 0.3)*	0.1 *(0.0 – 0.2)*	0.10	0.06	0.84
C10:0 (µg/mg)	0.0 *(0.0 – 0.4)*	0.0 *(0.0 – 0.0)*	0.0 *(0.0 – 0.2)*	0.62	0.96	0.87
C20:1 (µg/mg)	0.0 *(0.0 – 0.0)*	0.0 *(0.0 – 0.0)*	0.0 *(0.0 – 0.0)*	NA	NA	NA
ratio C18:0/C18:1 Δ9	11.6 *(10.1 – 13.2)*	10.0 *(9.0 – 11.8)*	9.5 *(7.2 – 9.9)*	0.13	**0.02**	0.63
ratio C16:0/C16:1 Δ6	2.2 *(1.9 – 2.6)*	2.1 *(1.7 – 2.5)*	2.3 *(2.0 – 3.5)*	0.45	0.55	0.08
ratio saturated/ unsaturated	1.9 *(1.7 – 2.1)*	1.8 *(1.6 – 2.1)*	1.9 *(1.6 – 2.2)*	0.85	0.91	0.31

Values described are medians *(interquartile ranges)*, with *p* values which are only exact for comparisons between groups. Significant *p* values are bolded. Bound fatty acid ratio values are listed at the end of the table and are separated by a dashed line.

Supplementary Table 4 Quantitative composition of face sebum versus scalp sebum using the novel sampling technique

**Lipid species**	**Forehead** (McGinley)	**Forehead** (In house)	**Scalp** (In house)
Free fatty acids	16 %	15 to 30 %	30 to 50 %
Squalene & wax esters	48 %	30 to 50 %	40 to 60 %
Cholesterol esters & free cholesterol	6 %	< 10 %	< 10 %
Glycerides	30 %	30 to 50 %	5 to 15 %

Quantitative composition of face sebum versus scalp sebum using the novel sampling technique in healthy volunteers (n=6), compared to previously-published forehead results from McGinley et *al* [20].
