# Supplementary material for: Exploration of scalp surface lipids reveals squalene peroxide as a potential actor in dandruff condition
Source: Arch Dermatol Res. 2016 Feb 3;308:153–63. doi: 10.1007/s00403-016-1623-1 (PMC4796319; doi:10.1007/s00403-016-1623-1)
Supplement: Supplementary file 3 — Supplementary material 3 (PDF 116 kb) [file 403_2016_1623_MOESM3_ESM.pdf]

Article Title: Exploration of scalp surface lipids reveals squalene peroxide as a potential actor in dandruff condition

Journal: Archives of Dermatological Research

Authors: Roland Jourdain, Alain Moga, Philippe Vingler, Charles el Rawadi, Florence Pouradier, Luc Souverain, Philippe Bastien, Nicolas Amalric, Lionel Breton

Corresponding Author: Roland Jourdain - L'OREAL Research & Innovation, Aulnay-sous-Bois, France - Email: rjourdain@rd.loreal.com

Supplementary Figures

Supplementary Figure 1 – MDA chromatographic profile

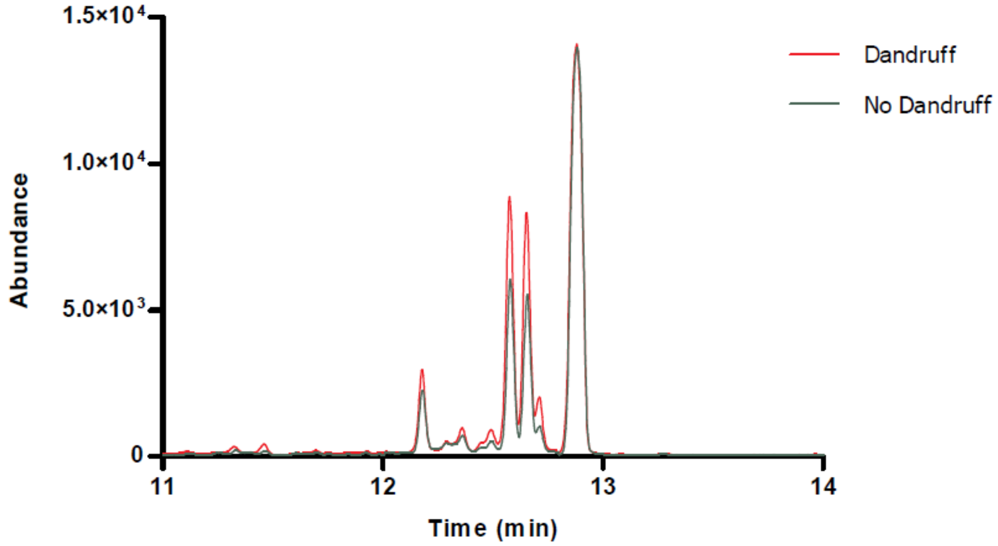

Supplementary Fig.1 Chromatographic MDA profile of both dandruff and non-dandruff zones from a given dandruff subject, indicating an elevated amount of MDA at the dandruff zone

Supplementary Figure 2 – Vitamin E chromatographic profile

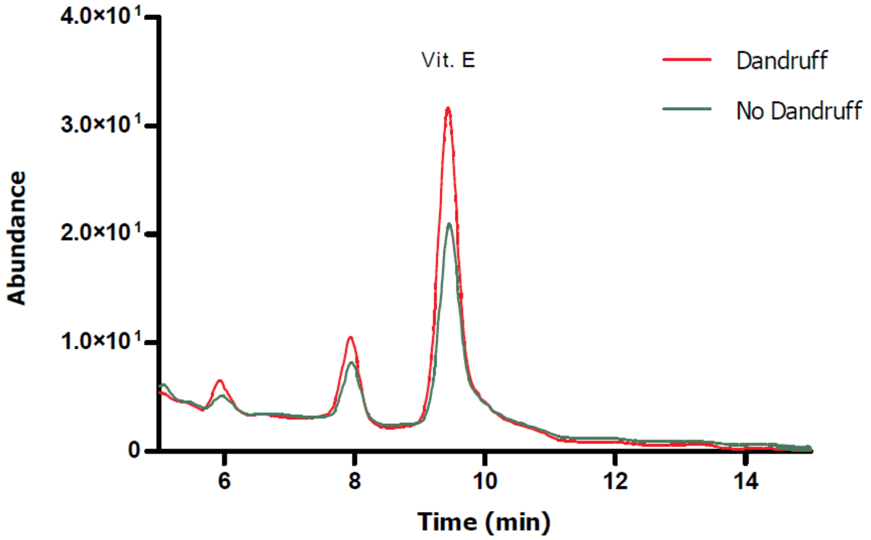

Supplementary Fig.2 Chromatographic profile of vitamin E of both dandruff and non-dandruff zones from a given dandruff subject, indicating an elevated amount of vitamin E at the dandruff zone

Supplementary Figure 3 – Free fatty acids chromatographic profile

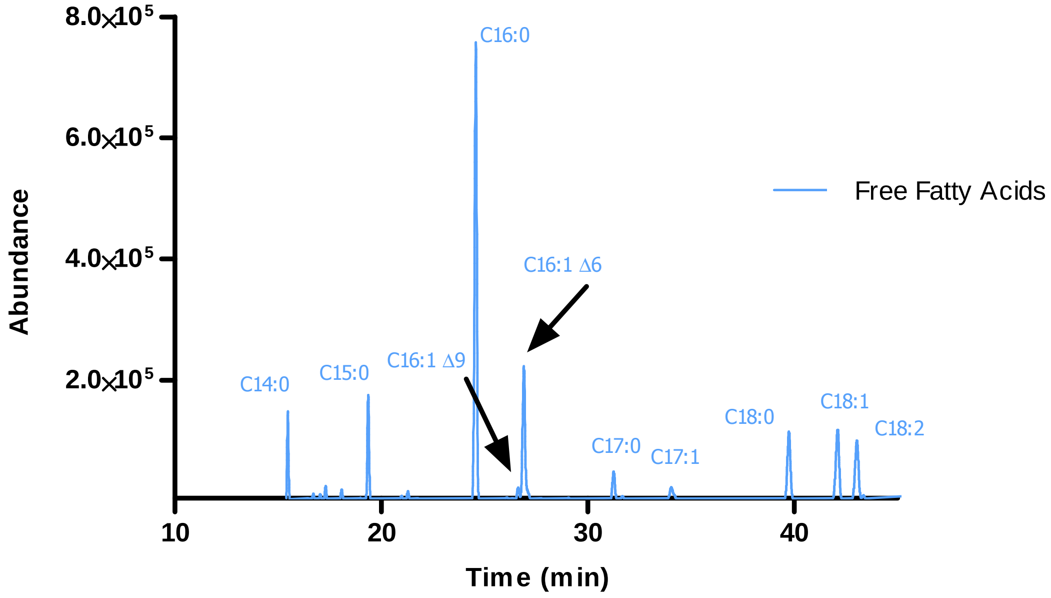

Supplementary Fig.3 Chromatographic profile of free fatty acids of the dandruff zone from a given dandruff subject, illustrating their separation according to chain length and number of double bonds
